# Evaluation of an End-of-Life Essentials Online Education Module on Chronic Complex Illness End-of-Life Care

**DOI:** 10.3390/healthcare8030297

**Published:** 2020-08-25

**Authors:** Deb Rawlings, Megan Winsall, Huahua Yin, Kim Devery, Deidre D. Morgan

**Affiliations:** Palliative & Supportive Services, Flinders University, Bedford Park, SA 5042, Australia; megan.winsall@flinders.edu.au (M.W.); huahua.yin@flinders.edu.au (H.Y.); kim.devery@flinders.edu.au (K.D.); deidre.morgan@flinders.edu.au (D.D.M.)

**Keywords:** end-of-life care, prognostic uncertainty, online learning, patient-centred care, communication, hope

## Abstract

Chronic complex illness/multimorbidity is a leading cause of death worldwide. Many people with chronic complex illnesses die in hospital, with the overall quality of end-of-life care requiring substantial improvement, necessitating an increase in the knowledge of the health professionals caring for them. End-of-Life-Essentials (EOLE) offers online education modules for health professionals working in acute hospitals, including one on chronic complex illness. A quantitative pre–post-evaluation analysis was undertaken on data from learners (*n* = 1489), who completed a questionnaire related to knowledge gained from module completion between December 2018 and November 2019. A qualitative post-evaluation analysis was also conducted using data on learner responses to a question posed between May and November 2019. Results showed a significant positive impact on learners’ knowledge, skill, attitude, and confidence in providing end-of-life care to patients living with chronic complex illness. The majority (82.9%, *n* = 900) intended to change their practice after module completion. A total of *n* = 559 qualitative comments were analysed thematically, with three major themes emerging: Patient centred care and care planning, Discussion of prognosis, and Valued communication skills. This evaluation has demonstrated that healthcare professionals could benefit from this education to improve quality of care of the dying.

## 1. Introduction

Slow dying and death in very old age caused by chronic complex illness is a relatively new phenomena in many countries [1]. The rise of public health measures, vaccinations, and medical science has not only changed the way we live, but also altered the way we die [2]. Globally, the leading causes of death today are ischaemic heart disease, stroke, chronic obstructive lung disease, and lower respiratory infections, and these have remained constant during the past decade [3]. Worldwide, deaths due to dementia have increased rapidly between 2000 and 2016, with dementia the 5th leading cause of death in 2016 [3]. Chronic complex conditions are the leading causes of death in Australia, and it is estimated that the number of Australians who die each year will double by 2040, making the equitable provision of end-of-life care critical [4].

A key challenge associated with chronic complex illness is prognostic uncertainty, that is, the uncertainty surrounding an individual’s prognosis and course of the illness [5]. Etkind et al. (2017) have used terms such as ambiguity, inconsistency, vagueness, unpredictability, lack of information, unfamiliarity, and complexity, to describe the impact of an uncertain prognosis. This uncertainty can be a source of distress for patients and families in hospitals [6], and is linked with end-of-life care outcomes [7]. Importantly, patients have shown improved quality of life when emotionally sensitive conversations pertaining to prognosis have been held, and information has been provided [6].

The Australian Commission on Safety and Quality in Health Care (ACSQHC) recommends that end of life should be identified 12 months prior to death in order to deliver safe, quality end-of-life care [8]. Delineating between an acute reversible deterioration and normal process of dying remains a major challenge in the delivery of quality end-of-life care. Many patients in Australia miss out on choice about the end of life, in part due to a healthcare system which defaults to curative treatments, unnecessary admissions to hospital, and a lack of response to end-of-life needs [9]. Provision of high-quality end-of-life care and identification of patients approaching end of life were identified as priorities for healthcare delivery and reported on in the Australian Government’s Productivity Report. This report also proposes a series of high-level reforms to strengthen the way services in all settings—hospitals, residential aged care, and at home—are provided to people [9].

A plethora of evidence supports the need for these important conversations to be held in a timely manner, but healthcare professionals (HCP) do not always recognise appropriate timing or have the knowledge, experience, or confidence to initiate these conversations [10]. HCP report that it is often easier to continue treatments than to talk and plan care with patients and families about the End-of Life [11]. Fear, denial, and unrealistic expectations by HCP can lead to a cascade of invasive treatments or referrals that result in poor end-of-life care [12]. This not only influences HCP clinical practice, but also health resource utilisation [13]. Medical advances globally have been very successful at treating, but not curing, chronic complex illnesses, thus, prolonging life. HCP can benefit from education, especially in relation to communication about end of life [5], to avoid providing care in a manner that does not enable patients to express their needs.

In 2016, the Australian Government Department of Health funded a series of palliative care program projects including End-of-Life Essentials (EOLE) (https://www.endoflifeessentials.com.au). EOLE provides clinician peer-reviewed and evidence-based online education and implementation tool kits for doctors, nurses, and allied health professionals who work in acute hospitals. The aim of the EOLE project is to increase Australian HCP knowledge and confidence in end-of-life care. EOLE is based on the work undertaken by the ACSQHC National Consensus Statement: essential elements for safe and high-quality end-of-life care [8]. The ACSQHC further developed their work to support health services to improve the quality of end-of-life care [8]. In 2019, the ACSQHC integrated end-of-life care standards into The National Safety and Quality Health Service Standards, second edition (NSQHS) to provide a nationally consistent quality assurance mechanism of the level of care consumers can expect from health service organisations.

The EOLE project has undertaken a range of data collection activities to explore and describe possible changes in the knowledge, skills, and behaviours of health professionals who engage with the EOLE online modules and resources. There are currently 10 EOLE modules, each with an embedded evaluation framework including routinely collected pre-test–post-test data and a free-text question posed at the end of each module [12]. The purpose of this study was to evaluate whether the EOLE education module ‘Chronic Complex Illness End-of-Life Care’ increased learner knowledge, and whether learners intended to change their clinical practice via the question: “How can you use ‘prognostic uncertainty’ in conversations about patients’ future goals of care?” This paper examines both quantitative and qualitative learner responses related to this module.

## 2. Materials and Methods

Module development was informed by an educational framework addressing quality matters, and in consultation with an Advisory Group, industry partners, and peer reviewed by Australian clinicians for currency and relevance [12]. Modules are based on the ACQSHC National Consensus Statement: essential elements for safe and high-quality end-of-life care [8].

‘Chronic Complex Illness End-of-Life Care’ (hereafter referred to as the chronic complex illness module) features in the suite of online education modules and explores:

- The impact of increased hospitalisation on people’s experiences and emotions in those living with chronic complex conditions.
- Clinical opportunities for end-of-life care along common disease trajectories in patients living with and dying from chronic complex conditions.
- The importance of ‘uncertainty’ in disease trajectories as a trigger in starting conversations in end-of-life care communication and in advance care planning.
- The potential for moral distress, self-care, and compassion associated with end-of-life care in a fast-paced environment.
- The opportunities for kindness and compassion in providing healthcare to people living with chronic complex disease.

The EOLE education modules are freely available and can be accessed by any health professional with an interest in end of life care in acute hospitals. Learners register to access the education and to date, there have been over 17,000 learners, the majority of whom are from Australia, but with many also registered from overseas, speaking to the relevance of the education globally. The education modules, as with other aspects of the EOLE project, are subject to routine, ongoing evaluation.

The chronic complex illness module has pre-test–post-test questions (quantitative measures) to assess changes in knowledge, skills, attitudes, and confidence as a result of taking the module, as well as a statement on whether learners intend to change their practice. A free-text question (qualitative measure) is also posed at the end of the module: “How can you use ‘prognostic uncertainty’ in conversations about patients’ future goals of care?”

### 2.1. Ethical Considerations

Ethical approval for the project was provided by Flinders University Social and Behavioural Research Ethics Committee (Project 7012). Learners are invited to complete pre-test and post-test questions with each module. Answers are not forced, and consent is implied with completion of the evaluation questions.

### 2.2. Data Collection and Analysis

Both the pre-test/post-test questions and the free-text questions are embedded in each EOLE module. The former are consistent across modules and the latter are relevant to the content of each module. All questions are voluntary, and learners can proceed through the education module and then exit without contributing to the evaluation. It is not compulsory for learners to provide responses for the surveys, but they need to click the ‘submit’ button to continue their module learning. Therefore, the response rate was calculated for the pre-evaluation survey and post-evaluation survey separately. The quantitative data and qualitative data are collected in and extracted from two different platforms (a learning management system and a research data management system) and as such, have no data linkage capabilities.

#### 2.2.1. Quantitative Data

Evaluation data for the quantitative arm were extracted for a 1-year period, from learners who completed pre- or post-evaluation questions for the chronic complex illness module from 4 December 2018 to 30 November 2019. In total, 1531 learners accessed the pre-evaluation survey, and 1466 learners answered at least one question, the response rate being 95.8%. Learners who did not provide any responses were removed from the pre-evaluation survey report. A total of 1150 learners accessed the post-evaluation survey, and 1099 learners answered at least one question, the response rate being 95.6%. Learners who did not provide any responses were removed from the post-evaluation survey report. The pre-evaluation responses and post-evaluation responses were deidentified and imported into SPSS separately, then, merged using the SPSS merge function.

In total, data from 1489 learners who completed at least one pre- or post-evaluation question were included for data analysis. The pre-evaluation statements were set out at the beginning of the chronic complex illness module under the header:

In thinking about providing end-of-life care for patients living with chronic complex illness:

- I have sufficient knowledge in providing end-of-life care;
- I am skilled in providing end-of-life care;
- I have a positive attitude towards end-of-life care;
- I am confident in my ability to provide good end-of-life care.

The post-evaluation questions about end-of-life care knowledge, skill, attitude, and confidence were identical to the pre-evaluation questions, with the addition of one extra statement: I intend to change my practice in end-of-life care. Learners were asked to select “strongly disagree”, “disagree”, “neutral”, “agree”, or “strongly agree” for each of the statements. Data analysis was conducted using SPSS version 25.00 (IBM, Armonk, NY, USA). Descriptive statistics were used for demographic information and the practice change statement and were summarised as frequency with percentage. The significance of differences in pre- and post-evaluation was tested by Wilcoxon Signed Ranks Tests. A value of *p* < 0.05 was considered statistically significant.

#### 2.2.2. Qualitative Data

Evaluation data for the qualitative arm were extracted for a 7-month period: 6 May 2019 to 30 November 2019. The post-module question, from which the qualitative data was taken, changed on 6 May to be specific to the chronic complex illness module, with a generic question in place prior to this. A total of 666 free-text learner statements responding to the chronic complex illness module open-ended question “How can you use ‘prognostic uncertainty’ in conversations about patients’ future goals of care?” were extracted from the EOLE learning platform. The data were cleaned, deduplicated, and deidentified, resulting in a total of 559 valid learner statements (1 statement per learner). The data were imported into NVivo 12, with each statement classed as an ‘open-ended’ response.

An inductive, ground-up approach to content analysis was conducted to identify key themes emerging from the data [14]. Thematic content analysis was chosen as the approach due to its suitability for analysing data on multifaceted healthcare phenomena [15], and also in allowing descriptive coding of the data alongside quantitative counts of the codes [14]. Researcher MW completed coding for all data (*n* = 559 learner statements) using NVivo 12 (version 12, QSR International Pty Ltd., Melbourne, Australia) and created a coding schema outlining each theme and subtheme. The data were coded line-by-line and similar concepts were grouped into themes and then, further into subthemes. This was an iterative process, with new themes and subthemes being created as coding commenced, and subsequent statements either coded into pre-existing subthemes, or into new subthemes where necessary [14]. In this way, codes were added and refined, in the process of ‘testing the fit’ of new data as the analysis progressed [16].

To assess the internal validity of the content analysis [15] and improve the reliability of the analytical approach [14], cross-verification coding was completed by researcher D.R., who coded a randomly generated set of 56 learner statements (10% of total statements) against researcher M.W.’s previously defined coding schema. For the first round, a total of 228 codes were assigned to the 56 statements, with agreement reached on 156 of the 228 codes (68.4%), a total of 72 coding discrepancies. In order to achieve a higher agreement rate, the researchers discussed the major sources of discrepancy within the coding schema, including a thorough overview of each theme description. Two of the existing themes were restructured/renamed for clarity. One additional subtheme was also created upon discussion between the researchers; however, no additional subthemes emerged.

A second round of cross-verification was completed by researcher D.R., with a new set of randomly generated learner statements. A total of 212 codes were assigned to the 56 statements, with agreement reached on 146 of the 212 codes (68.9%), similar to previous coding. A further discussion was held to ascertain discrepancies and subthemes were combined as a result. After these modifications to the coding schema, a third cross-verification round of 56 statements was carried out. A total of 236 codes were assigned to the 56 statements and the independent coders matched on 196 of the 236 (83.1%), with 40 coding discrepancies across the 56 statements. Cohen’s Kappa was calculated to determine the proportion of inter-rater agreement which would have been expected from chance alone. The Cohen’s Kappa for inter-rater agreement was 0.759, *p* < 0.000, indicating a substantial level of agreement between the two independent researchers [17].

Descriptions of each theme and subtheme, as well as exemplar quotes and quantitative summaries, are presented in tables [18].

## 3. Results

### 3.1. Quantitative Results

#### 3.1.1. Demographics

**Figure 1:**
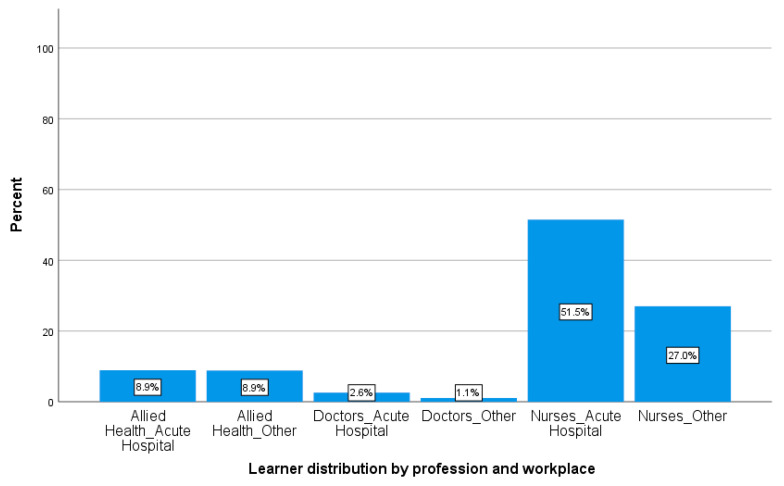
Learner distribution by profession and workplace (*n* = 1489).

The majority of learners were nurses (78.5%, *n* = 1169), followed by allied health professionals (17.8%, *n* = 265). The majority of learners were from acute hospitals (63.1%, *n* = 939) (see Figure 1).

#### 3.1.2. Impact on Learners’ Knowledge, Skill, Attitude, and Confidence

There were significant improvements in learners’ knowledge, skill, attitude, and confidence in providing end-of-life care to patients living with chronic complex illness after completing the EOLE chronic complex illness module. Wilcoxon Signed Ranks Test indicated that the post-evaluation ranks of knowledge, skill, attitude, and confidence were statistically significantly higher than the pre-evaluation ranks of knowledge, skill, attitude, and confidence (see Table 1).

#### 3.1.3. Intention to Change Clinical Practice

**Figure 2:**
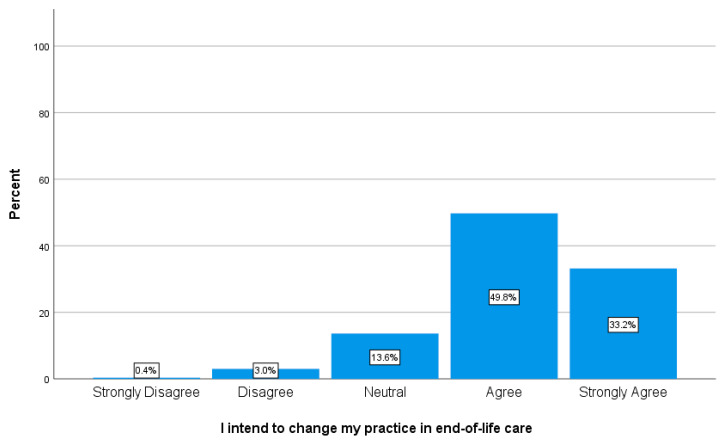
Intention to practice change in providing end-of-life care for patients living with chronic complex illness (*n* = 1085).

The majority of learners agreed/strongly agreed (49.8%, *n* = 540/33.2%, *n* = 360) that they intended to change their practice in providing end-of-life care to patients living with chronic complex illness (see Figure 2).

### 3.2. Qualitative Results

Eleven subthemes from 559 learner statements were organised into three overarching themes: Patient-centred care and care planning, Discussion of prognosis, and Valued communication skills.

#### 3.2.1. Patient-Centred Care and Care Planning

This theme related to the importance of placing the patient at the centre of the care plan and incorporating the goals and wishes of the patient and their family into this care. In total, 366 (65.5%) learner statements related to this theme.

#### 3.2.2. Discussion of Prognosis

This theme related to discussing the patient’s medical condition, prognosis, and disease trajectory, including any uncertainty around the amount of time they may have left, and providing accurate medical information on illness status to the patient and their family. In total, 314 (56.2%) learner statements related to this theme (Table 2).

#### 3.2.3. Valued Communication Skills

This theme related to specific communication skills and techniques that are important for HCP when caring for patients and discussing future goals of care and prognostic uncertainty. In total, 209 (37.4%) learner statements related to this theme (Table 3).

## 4. Discussion

Many expected patient deaths take place in hospital, with the overall quality of end-of-life care in these settings requiring substantial improvement [19]. In order to improve end-of-life care, HCP require upskilling in both knowledge and abilities [12]. Completion of the chronic complex illness module led to a self-reported significant, positive influence on the knowledge, skill, attitude, and confidence of learners in provision of end-of-life care to patients living with chronic complex illness. The majority of learners (over 80%) also indicated that they would change their clinical practice when caring for those with chronic complex illness (see Figure 2). This in itself can be viewed in a positive light, with learners acknowledging that they can, and perhaps need to, change the care that they are currently providing. Intent to change practice has long been contended as a way of predicting actual behaviour change (including in health professionals), although with acknowledgement that direct observation of change is the gold standard and that other methods of measurement do involve compromise [20].

Online learning has become increasingly popular with health professionals, is at least as effective as face-to-face education, and as our findings demonstrate, can help to improve knowledge in certain areas, including end of life [21]. Analysis of free-text responses lends support to the quantitative findings and identifies three key areas prioritised by HCP. These are patient-centred care, acknowledging and communicating prognostic uncertainty, and the importance of skilled communication.

### 4.1. Patient-Centred Care

Patient centred care was highlighted by learners as an important aspect of care, and that including the patient and family in the development of care plans supports this approach. Subthemes comprised inclusive discussions and providing advice to patients and families, patient comfort and quality of life, a team-based approach, and to focus on the present.

Patient-centred care is complex, multifaceted, and hard to define [22]. Individualised or patient-centred care has been identified as an essential component of a safe and high-quality health system in Australia [23]. Patient-centred care, as highlighted in our study, includes the basic tenets of patients and/or family involvement in decision-making [24], of open communication between patients, families, and HCP [25], of sharing power and responsibility, and of accepting patients’ life choices [22]. Patients’ values, priorities, and goals are considered central to this care planning process, although patients do need to have an understanding of their illness to be able to participate fully in any discussions [26]. Gluyas et al. (2015) have highlighted barriers to patient-centred care, including lack of continuity and fragmentation of care, as well as the power differential between patients and HCP. However, they note that patient-centred care, if implemented well, has been reported to improve outcomes for patients and increase satisfaction with care [25].

‘Goals of care’ is a term that is used increasingly in the end-of-life literature, often in the context of patient-centred care and care planning; however, the term can be interpreted in different ways [26]. It is often used to cover discussions about treatment intent and planning end stage care with patients [27]. The term is often confused with Advance Care Planning (ACP) or discussions that relate to treatment limitation [28], so establishing a common understanding of what discussing ‘goals of care’ entails is important [22]. Goals of care conversations are usually driven by the patient’s and family’s hopes, fears, expectations, and preferences for information, with treatment and care plan decisions often based on discussion and consideration of patient comfort and quality of life [27]. Supporting patient comfort and quality of life also emerged as subthemes in our study and were considered important by HCP. These findings are supported by Etkind and colleagues (2017) who found that quality of life was often considered a patient priority in decision making. These authors also describe the theme “temporal focus: facing an uncertain future” which relates to the dichotomy of patients focusing on either their present or future health dependent upon current circumstance [5] (p. 175). This aligns with our study findings, where learners encouraged patients to focus on today rather than on an uncertain future (see Table 4).

### 4.2. Acknowledging and Communicating Prognostic Uncertainty

The second key concept emerging from the content analysis was that of proactively discussing the prognosis. This theme centred on the importance of having discussions with the patient about their medical condition and prognosis, including any uncertainty around the amount of time they may have left before they die. The provision of accurate information to the patient and their family was valued by respondents, as was determining what the patient wanted to know and then, checking their understanding

Goals of care discussions at end of life must involve the consideration of a patients’ prognosis. Our learners identified the need to acknowledge uncertainty in relation to time until death. This is an important concept when considering prognostication, especially in those with non-malignant conditions, as illness trajectory is less certain than in most malignant diseases [29]. Krawczyk and Gallagher (2016), in their study with bereaved family members, focussed in part on prognostic communication [30], and identified six themes on this aspect: lack of awareness the patient was sick enough to die, lack of communication about possible prognosis, dissonance between probable outcome of care and ongoing treatments, inappropriate use of euphemisms, provision of false hope, and suspicion of malfeasance. Families who reported that discussions had been held about prognostic uncertainty had increased satisfaction with care [30]. This supports our findings that learners consider acknowledging uncertainty and providing information, checking what the patient understands, and asking what they want to know, as key to prognostic communication.

Anderson et al. (2019) found that ‘highlighting deterioration’ was an important element, which involves detailing past and current health problems to emphasise end of life was approaching [24]. The promise of ongoing, continuing care was underlined as essential [24], which is consistent with our findings. Another significant element of these conversations around prognosis gleaned from the current study was the need to find out what the patient knows or understands of their current and future health, and what more they want to know. A study by Etkind and colleagues (2017) found that many participants understood the big picture but did not comprehend what the future held. Some wanted to know everything and others wanted no information at all [5]. A number of learners in our study stated that it was important to check what the patient understood about their potential illness progression, and how much they wanted to know before embarking in conversations about this. This is reinforced in a study by van Vliet et al. (2019) when talking with patients with advanced cancer, where a balancing act was described in preparing patients for uncertain or poor outcomes while also trying to offer some hope, with the provision of accurate information key to future planning [31].

### 4.3. The Importance of Skilled Communication

The third key concept emerging from the content analysis was the use of skilled communication. This theme highlighted specific communication skills and techniques that are important for health professionals when caring for patients and discussing future goals of care, where prognostic uncertainty is involved. Emerging also were the subthemes of honesty, being supportive, hope, and the use of open-ended questions.

Appropriately skilled communication with people who have chronic complex illnesses is vital and requires great thought and consideration, especially in how prognostic uncertainty is conveyed [13]. The communication skills subthemes that emerged in the current study were honesty, being supportive, hope, and employing open-ended questions.

The need for effective communication with patients and families in end-of-life care has been reported in hospitals over many years [19,32]. Effective communication requires open and honest conversations that contribute to respectful and compassionate care and has been described as important from the patients’ perspective [33,34]. The concept of honesty [24] was reported by learners in the current study as important, as was empathy [35], listening [12], support, reassurance, and kindness, with such caring behaviour a vehicle to instil hope [36]. Hope has been described as a “powerful and vital coping mechanism for a person who is nearing the end of their life” [37] (pp. 4), however, when conveying hope to patients, clinicians need to strike a balance between realism and optimism [38]. It is important not to take away hope [36,37], but also not to give false hope [30]. One Dutch study looking at physician–patient communication found that participants wanted to be given realistic information with empathy, rather than unrealistic/hopeful information [39]; an important concept highlighted elsewhere, with a lack of empathy seen when oncologists did not pick up on a patient’s emotions [31]. Communication skills, such as those required in end-of-life conversations and when communicating uncertainty, can be improved through training, as well as normalising and embedding end-of-life discussions into routine clinical practice [10].

### 4.4. Implications for Future Research and Patient Care

There are increasing numbers of people dying in acute hospitals who will require care by HCP, many of whom could benefit from the EOLE education to improve their quality of care. This study highlights the importance of skilled communication with patients and families, embedded into patient-centred care. Prognostication and openly, honestly acknowledging and discussing prognostic uncertainty are important, the key component being how professionals communicate, e.g., the use of hope and empathy when providing information.

Further evaluations of the other EOLE modules to identify the benefits of ongoing education in this area are planned. Other work that intends to explore barriers and enablers to implementation in practice, via a systematic literature review and qualitative interviews with learners, is underway. Further enquiry into changes in practice that have been undertaken (rather than intended) may also be possible. Another consideration is that of whether the education modules impact different health disciplines in different ways.

The development and evaluation of training that focusses on practical communication strategies requires prioritisation. This training can be used by HCP when speaking with patients with chronic illnesses about their prognosis and goals of care. Training could focus on how best to use open-ended language, empathy, hope, and honesty to enhance the care of patients who are facing the end of their life [34].

### 4.5. Strengths and Limitations

The number of self-selected, proactive, and motivated learners who responded to the embedded questions in the modules is a strength of this study. However, while the pre–post approach enables the measure of change in large numbers, the self-selected nature of the learners is a limitation as it does not tell us how many health professionals do not pursue this education. It is possible that responses by non-responders to the evaluation component of the modules would show different perspectives to those who have participated in the evaluation and provide alternate viewpoints. There is also a limitation in responding to a single question without any further critical insight into what practice change would look like [12].

The self-report nature of the evaluation and study design did not allow us to ascertain if behaviour change did indeed take place. As a self-report, this evaluation has not been validated or measured against other standardised assessments. Therefore, it is not possible to determine effectiveness of the module learning on practice change, with self-assessment not always correlating with objective assessment by others [40]. Self-reported measures may also vary based on years of experience, which is also an unmeasured factor here.

To be considered also is the inability to link qualitative and quantitative data due to the incompatibility of the two data capture platforms.

A further consideration is that the majority of learners are nurses and their responses will ultimately drive the results, although learner profession demographics do reflect that of the healthcare workforce, with nurses the largest group of providers.

## 5. Conclusions

The evaluation of the EOLE chronic complex illness module showed a statistical improvement in self-reported learners’ knowledge, skill, attitude, and confidence following education module completion. The majority of learners who completed the module also reported that they intended to change their clinical practice when providing end-of-life care to patients living with chronic complex illness; however, further understanding of what constitutes an ’intention to change practice’, and how this is achieved, is required. Improved satisfaction and patient outcomes can potentially be achieved with provision of patient-centred care, targeted goals of care discussions, and discussions around prognostic uncertainty. The key to effectively addressing prognostic uncertainty is the way in which prognostic information is communicated to patients, incorporating empathy, hope, honesty, and openness of discussion. This evaluation has demonstrated that healthcare professionals could benefit from this education to improve quality of care of the dying.

## Figures and Tables

**Table 1:** Learners’ knowledge, skill, attitude, and confidence in providing end-of-life care to patients living with chronic complex illness.

Statements	Pre-Evaluation Mean ± SD	Post-Evaluation Mean ± SD	Wilcoxon (Z)	*p* Value	Effect Size
I have sufficient knowledge in providing end-of-life care	3.53 ± 0.95	4.03 ± 0.74	−16.748	<0.001	−0.362
I am skilled in providing end-of-life care	3.50 ± 1.0	3.95 ± 0.79	−15.573	<0.001	−0.339
I have a positive attitude towards end-of-life care	4.17 ± 0.75	4.36 ± 0.64	−10.130	<0.001	−0.220
I am confident in my ability to provide good end-of-life care	3.76 ± 0.89	4.10 ± 0.73	−13.993	<0.001	−0.304

Scores reported are average ratings on a five-point Likert scale: 1 = strongly disagree; 2 = disagree; 3 = neutral; 4 = agree; 5 = strongly agree. Statistical significance was deemed at *p* < 0.05.

**Table 2:** Discussion of prognosis subtheme descriptions.

Subtheme	Description	No (%) Learners	Example Quotes
Acknowledge the uncertainty and give information on prognosis	This pertains to providing the patient and family with accurate information on their current condition, illness, or disease trajectory, and seeking further clarification from other medical staff if required, in order to answer patient questions. HCP also acknowledged to the patient and family that there was some uncertainty about the patient’s disease progression and amount of time they may have left prior to death. They also discussed the possibility that patients are approaching their end of life.	280 (50.1%) learner statements related to this subtheme.	*“Admit we don’t know what the future may hold with your illness”* *“Acknowledge that dying is a possibility”* *“By including conversations regarding prognostic uncertainty, we can often alleviate some of the distress regarding end of life”* *“Discuss facts and diagnostic test results”*
Determine what the patient wants to know	This pertains to the importance of HCP asking the patient and family how much information they want to be given about their illness and prognosis. Some patients may want to know more than others, but what is most important is establishing this at the outset.	51 (9.1%) learner statements related to this subtheme.	*“…the patient can still express preferences about the amount of knowledge they would like about what to expect in the course of their illness.”* *“Would they like more information about how the illness will most likely progress or not?”*
Check patient’s understanding	This pertains to HCP asking the patient how much they currently know and understand about their medical condition, disease trajectory, and prognosis.	31 (5.5%) learner statements related to this subtheme.	*“It is important to simply ask the patient about their current condition and whether they understand the situation.”* *“consider the disease process and the patient and families understanding of that process.”*

**Table 3:** Valued communication skills subtheme descriptions.

Subtheme	Description	No (%) Learners	Example Quotes
Honesty	This pertains to the importance of being honest and telling the truth, i.e., ensuring discussions around goals of care and prognostic uncertainty are done so with honesty on the part of the HCP.	108 (19.3%) learner statements related to this subtheme.	*“Always be honest with the patient”* *“Be honest and realist with them.”* *“Discussing all the possible outcomes very honestly.”*
Being supportive	This pertains to providing support and reassurance to the patient and family (e.g., emotional support, taking the time to be with the patient), as well as being friendly, warm, and kind (e.g., through acts of kindness). Included also were comments around the use of empathy, i.e., recognising and acknowledging emotions, and the importance of simply listening to all patient concerns.	106 (19.0%) learner statements related to this subtheme.	*“Be present and listen to concerns and emotions try not to fix situation or be to clinically focused treat the patient holistically, spiritually, emotionally and physically.”* *“Demonstrate your care and compassion through your kindness and patience in providing care.”*
Hope	This pertains to the importance of the HCP maintaining a sense of hope and positivity, and of not providing ‘false hope’.	33 (5.9%) learner statements related to this subtheme.	*“Instil hope, but not false hope.”* *“Compassionately balancing hope while also being honest, open and clear.”* *“We can plan for the worst and still hope for the best”*
Open-ended questions and discussion	This pertains to the importance of keeping communication between the patient, family, and HCP as open as possible, in order to encourage questions and continued discussion.	31 (5.5%) learner statements related to this subtheme.	*“Keep the communication lines open as they learn together what the future may look like.”* *“Through open dialogue patients can find encouragement and hopefully peace of mind to face the future whatever that holds.”*

**Table 4:** Patient-centred care and care planning subtheme descriptions.

Subtheme	Description	No (%) Learners	Example Quotes
Discuss, advise, and include patients and their families in end-of-life and future care planning	This pertains to ways in which HCP inform the patient and family about the range of supportive services, and care plan/treatment options that are available to them, as well as facilitating and encouraging open discussion about death, dying, and care planning for end of life. This included enabling the patient to express their goals and wishes, and what was most important to them when it came to their care and the time they had left. Developing a plan of care that enabled the patient to achieve their specific goals and wishes and involving the patient and family in the care planning process was very important.	332 (59.4%) learner statements related to this subtheme.	*“Informing the patient and their family about various options for future care”* *“Acknowledging that it is a time to start thinking and planning for End of Life Care”* *“Tease out what’s most important to the patient as those things can become goals of care.”*
Patient comfort and quality of life	This pertains to HCP ensuring and prioritising patient comfort care, by providing pain relief and management of other symptoms to maximise their quality of life for the time they had left.	54 (9.7%) learner statements related to this subtheme.	*“As a nurse I would make sure the patient is comfortable having no pain”* *“Focus on managing symptoms and pain to maximise quality of life for however long that may be”*
Multi-disciplinary/ team-based approach	This pertains to multidisciplinary staff members collaborating or using a team-based approach when discussing patient care plans together with the patient or relying on each other for information.	28 (5.0%) learner statements related to this subtheme.	*“It is a collaborative approach between health care professionals”* *“Refer patient and/or family to other members of the multidisciplinary team as appropriate”*
Focus on the present	HCP described encouraging the patient to focus on the day-to-day and to take each day as it comes as they approach their end of life.	20 (3.6%) learner statements related to this subtheme.	*“Have short term goals to achieve, take each day as it comes”* *“The conversation needs to focus on the near future and not on the late future.”*
